# Nano-Drug Delivery Systems in Oral Cancer Therapy: Recent Developments and Prospective

**DOI:** 10.3390/pharmaceutics16010007

**Published:** 2023-12-19

**Authors:** Yun Zhang, Yongjia Wu, Hongjiang Du, Zhiyong Li, Xiaofeng Bai, Yange Wu, Huimin Li, Mengqi Zhou, Yifeng Cao, Xuepeng Chen

**Affiliations:** 1 Stomatology Hospital, School of Stomatology, Zhejiang University School of Medicine, Clinical Research Center for Oral Diseases of Zhejiang Province, Key Laboratory of Oral Biomedical Research of Zhejiang Province, Cancer Center of Zhejiang University, Hangzhou 310006, China; 12318614@zju.edu.cn (Y.Z.); yogurtwu@zju.edu.cn (Y.W.); hxlzy2002@zju.edu.cn (Z.L.); baixf@zju.edu.cn (X.B.); wuyange@zju.edu.cn (Y.W.); 22218801@zju.edu.cn (H.L.); zhoumengqi@zjkq.com.cn (M.Z.); 2 Department of Stomatology, The Second Affiliated Hospital of Zhejiang Chinese Medical University, Hangzhou 310005, China; 20014004@zcmu.edu.cn; 3 Department of Chemical and Biological Engineering, Zhejiang University, Hangzhou 310058, China

**Keywords:** oral cancer, nanomaterial, nanoparticle, nano-drug delivery system, targeting

## Abstract

Oral cancer (OC), characterized by malignant tumors in the mouth, is one of the most prevalent malignancies worldwide. Chemotherapy is a commonly used treatment for OC; however, it often leads to severe side effects on human bodies. In recent years, nanotechnology has emerged as a promising solution for managing OC using nanomaterials and nanoparticles (NPs). Nano-drug delivery systems (nano-DDSs) that employ various NPs as nanocarriers have been extensively developed to enhance current OC therapies by achieving controlled drug release and targeted drug delivery. Through searching and analyzing relevant research literature, it was found that certain nano-DDSs can improve the therapeutic effect of drugs by enhancing drug accumulation in tumor tissues. Furthermore, they can achieve targeted delivery and controlled release of drugs through adjustments in particle size, surface functionalization, and drug encapsulation technology of nano-DDSs. The application of nano-DDSs provides a new tool and strategy for OC therapy, offering personalized treatment options for OC patients by enhancing drug delivery, reducing toxic side effects, and improving therapeutic outcomes. However, the use of nano-DDSs in OC therapy still faces challenges such as toxicity, precise targeting, biodegradability, and satisfying drug-release kinetics. Overall, this review evaluates the potential and limitations of different nano-DDSs in OC therapy, focusing on their components, mechanisms of action, and laboratory therapeutic effects, aiming to provide insights into understanding, designing, and developing more effective and safer nano-DDSs. Future studies should focus on addressing these issues to further advance the application and development of nano-DDSs in OC therapy.

## 1. Introduction

Oral cancer (OC), explicitly affecting the oral cavity and oropharynx, is classified as a subtype of head and neck cancer. It is characterized by the presence of malignant tumors in various tissues, including the lips, tongue, palate, floor of the mouth, hypopharynx, oropharynx, larynx, alveolar mucosa, buccal mucosa, gingiva, or a combination of these [1,2,3,4,5,6]. The most frequently occurring OC cases are lip and oral cavity cancers. Oral squamous cell carcinoma (OSCC) is the predominant type of OC, accounting for 90% of OC cases and ranking among the 15 most common cancers worldwide. OSCC is highly dangerous, with a high morbidity rate, malignancy, and poor prognosis. Treating OSCC is challenging due to its high recurrence rate and tendency for lymph node metastasis [3].

Excessive alcohol and tobacco use, biological factors such as human papillomavirus (HPV), syphilis, oro-dental factors, betel chewing, nutritional deficits, and viruses are potential risk factors for OC. Patients with OC may experience early symptoms such as non-healing ulcers, intraoral bleeding, leukoplakia, erythroplakia, oral submucous fibrosis, and abnormal lumps in the mouth [4]. However, during the early stages of OC, many patients may not experience noticeable symptoms or signs of deterioration. This can lead to a delayed diagnosis and, in some cases, may result in the cancer progressing to an advanced, incurable stage, ultimately leading to increased medical expenses for the patient [5,6]. Globally, the survival rates for OC patients over five years range from 45% to 72% [1,2]. The survival rates gradually decrease with the delay of disease detection, with the five-year survival rate approaching 80% when OC is detected at an early stage but dropping to less than 20% when it is diagnosed at a late stage [7].

For several decades, the primary treatments for OC have been surgery, chemotherapy, radiation therapy (RT), and combination therapy. However, these treatments often have severe side effects and toxicity [8,9,10]. OC surgery can result in impairment or changes in breathing, swallowing, speaking, and other functions due to the removal of some oral tissues. It can also cause pain, swelling, bruising, xerostomia, infection, and bumps or scars on the face or neck [11]. RT can lead to side effects, such as oral mucosal inflammation, hypofunction of salivary glands, dysphagia, oral infections, nausea, vomiting, and skin reaction [12]. Chemotherapy involves the use of highly toxic drugs like cisplatin (CDDP), paclitaxel (PTX), 5-fluorouracil (5-FU), doxorubicin (DOX), methotrexate, cetuximab, and docetaxel (DTX), which can cause ulcers, mucositis, xerostomia, and damage to the skin, hair, blood, and kidneys [13,14,15,16,17,18,19]. Although low-toxic therapies, such as light stimulus-responsive therapies, including photodynamic therapy (PDT) and photothermal therapy (PTT) [20,21], as well as immunotherapy [22,23], have been studied for OC treatment, they have not been mature in clinical practice.

The clinical manifestations and treatment effects on OC patients can cause severe impacts on their lives, including adverse psychosocial effects from various aspects, the decline and loss of aesthetic and oral function, and the ensuing physical changes. Moreover, the economic cost of cancer treatment can increase the burden of OC patients and significantly impact their quality of life [24]. Therefore, there is an urgent need for innovative therapeutic strategies that prioritize bioavailability and precise drug delivery while minimizing harm to healthy cells or tissues [25].

In recent years, nanotechnology has played a crucial role in this regard. Various nanoparticles (NPs) have been developed to detect, diagnose, and treat OC [26,27] while minimizing damage to healthy cells during treatment [1,28,29,30]. Nanomaterials have emerged as vital tools in the development of new and effective therapies for OC [29] since they can be directly used as antitumor drugs [3,31,32,33,34,35,36,37] or utilized to form nano-drug delivery systems (nano-DDSs) for the administration of antitumor drugs.

DDSs are commonly used to address issues such as low solubility, poor absorption, poor permeability, inappropriate size, instability, and first-pass metabolism of chemotherapy drugs. Conventional DDSs like oral tablets, capsules, and injections, have been widely used in clinical settings to control drug release and ensure targeted delivery, resulting in improved pharmacokinetics, sustained bioavailability, and enhanced distribution at the primary tumor site [1,5,38,39,40]. In addition, the manufacturing process for conventional DDSs is well-established and cost-effective. However, many conventional DDSs lack the ability to specifically target diseased cells or tissues, leading to potential off-target effects and low accumulation at the desired sites. Individual differences in absorption and metabolism can also affect the bioavailability of drugs in conventional DDSs, resulting in inconsistent treatment outcomes. Additionally, the poor solubility of certain drugs poses challenges in formulating them into effective conventional DDSs. In contrast, nano-DDSs offer various advantages over conventional DDSs. They can be designed to target specific cells or tissues, increase drug delivery efficiency, minimize off-target effects, and can also improve the solubility of poorly water-soluble drugs, enabling the delivery of a wider range of therapeutic compounds [30,41,42]. In addition, due to the high permeability of micro-vessels in tumor tissues and the poor lymphatic drainage, NPs can take advantage of the enhanced permeability and retention (EPR) effects, swiftly entering tumor tissues and accumulating at tumor sites, allowing for sustained and controlled release [43]. Despite their benefits, some nanomaterials used in nano-DDSs may have toxicity issues, and their effective clearance and biodegradation in the body may present challenges, potentially leading to long-term accumulation and effects. Therefore, a comprehensive safety assessment is required. Furthermore, the manufacturing and scaling of nano-DDSs are technically challenging and require specialized equipment and expertise. So far, many NPs have been utilized as carriers for antitumor chemotherapy drugs and other therapeutic agents. This review provides detailed descriptions of nanomaterials and NPs used for antitumor drug delivery in OC therapy, aiming to assist researchers in understanding, designing, and developing more effective and safer nano-DDSs.

## 2. Targeting Methods for Nano-DDS in OC Therapy

**Figure 1:**
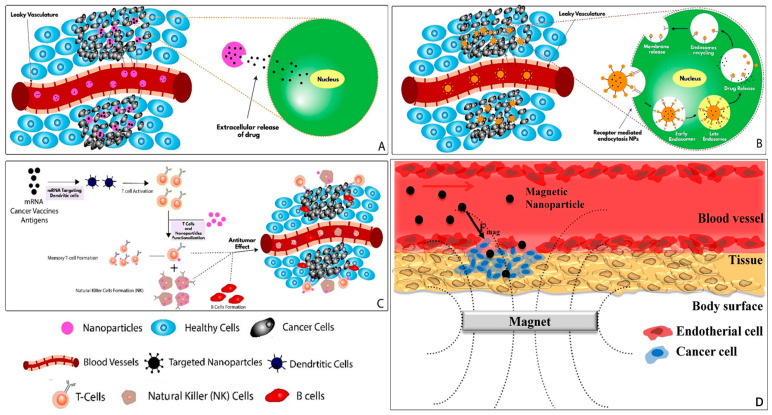
(**A**) Passive targeting refers to releasing drugs into tumor cells from nanoparticles (NPs) by the enhanced permeability and retention (EPR) effect through leaky vasculature. The drugs are released into the extracellular matrix from NPs and penetrate the tumor cells. (**B**) Active targeting: receptors on tumor cells can be targeted and bound with a specific targeting ligand on the surface of NPs, allowing the drugs to be released directly into the tumor cells. This method leads to a more significant accumulation of the drugs and uptake by the cells through the receptor-mediated endocytosis pathway. (**C**) Immune targeting: instead of administering drugs directly to the tumor, new immune-based therapy induces antitumor T cells, natural killer cells, and B cells. The treatment involves nucleic acid-based nano-vaccines that target dendritic cells, activating antibody cells and programming tumor cell death in turn. Reproduced with permission from ref. [5] (Copyright 2023 Elsevier). (**D**) Magnetic drug targeting with an external magnet. Reproduced with permission from ref. [45]. (Copyright 2010 Elsevier).

Targeted nano-DDS is a technology that allows drugs to be delivered precisely to the targeted organs, cells, or molecules [44], thereby safeguarding healthy cells and minimizing the side effects of loaded drugs, making it a practical approach for treating OC. Targeted drug delivery can be categorized as passive, active, immune, and magnetic targeting based on the targeting methods (Figure 1).

### 2.1. Passive Targeting

Passive targeting relies on the EPR effect of tumor tissue. The neovascularization in tumor tissue allows small-diameter nanocarriers to easily pass through the blood vessel wall and reach the tumor tissue with higher permeability compared to normal tissue. Nanocarriers are modified and assembled with drugs to increase their stability in plasma and extend their circulation time in the blood. This increases the chances of the nanocarriers aggregating in tumor tissues. The drugs are then delivered to the tumor cells through extracellular diffusion, which enhances the distribution and retention time of the drugs [44] (Figure 1A).

To achieve a better EPR effect, the nano-DDS must maintain stability in plasma without leakage and have a long blood circulation time to increase the chance of aggregation in tumor tissues [46] while also being able to penetrate deeply into the tumor. However, achieving long blood circulation and deep penetration into the tumor can be challenging, as these requirements may contradict each other. To achieve long circulation in the blood, the nano-DDS must possess a slightly larger particle size (such as 50~200 nm, preferably ca. 100 nm) [47] with a hydrophilic modification on the surface and be negatively charged [48]. On the other hand, for optimal uptake by tumor cells and deep penetration into the tumor tissue, nano-DDS should have a smaller size (such as <50 nm) and a positively charged surface after diffusion from capillaries to the tumor site [49]. In addition, the shape of the nano-DDS also plays a role in enhancing the EPR effect. By rationally designing the nano-DDS through size variation [50,51], shape modification [52], surface charge reversion [53], and other factors, the EPR effect can be improved.

### 2.2. Active Targeting

For active targeting, the surface of nanocarriers is typically modified with specific ligands, such as aptamers, peptide chains, and antibodies. By recognizing and binding to receptors on tumor cells and in the tumor microenvironment (TME), the nanocarriers deliver drugs to the intended sites, effectively enhancing the precision and efficacy of drugs and minimizing toxic side effects of drugs on normal tissues [44,54,55] (Figure 1B).

In targeted tumor therapy for OC, various sites such as tumor vessels, interstitial fluid and extracellular matrix, tumor matrix cells, tumor cells, related dendritic synaptic cells, and tumor stem cells can be targeted using nano-DDSs [28]. Depending on the characteristics of the tumors, different target sites can be selected in clinical treatment and nanocarriers can be modified accordingly. The identified overexpressed receptors in OC cells are listed in Table 1.

### 2.3. Immune Targeting

Cancer cells have developed various strategies to evade the immune system, such as reducing the expression of cell surface antigens, secreting antigens that inactivate the immune system, and stimulating the TME to release immune-suppressing chemicals that promote tumor growth. Cancer immune targeting is a treatment method that aims to activate the host’s immune system using foreign materials, including both active and passive immunity approaches (Figure 1C). Active immunotherapy involves stimulating the production of antibodies that specifically target and eliminate tumor cells by administering vaccines containing tumor antigens into the patient’s body. On the other hand, passive immunity involves using highly specific antibodies as carriers to introduce foreign substances with antitumor effects into the body. These antibodies target specific receptors on the surface of tumor cells, thereby enhancing the immune response. Nano-DDSs offer a promising approach in the field of OC immunotherapy, directly targeting cancer cells and helping facilitate the intracellular penetration of therapeutic agents, potentially minimizing autoimmune side effects and reducing treatment costs [78,79,80].

### 2.4. Magnetic Targeting

Magnetic NPs are inorganic materials that can be easily manipulated using external magnetic field gradients, allowing for precise targeting of desired sites [45,81] (Figure 1D). Magnetic targeting is a technique used in cancer therapy where magnetic NPs are delivered to tumor cells and concentrated in the lesion area using external magnets. This method enhances the effectiveness of chemotherapy drugs that are administered along with magnetic NPs. Furthermore, magnetic NPs also possess magnetic hyperthermia properties, which can be utilized in an alternating magnetic field (AMF) to kill tumor cells.

## 3. Nano-DDSs in OC Therapy

Nano-DDSs employ NPs as drug carriers, leveraging the enhanced EPR effect of passive targeting and other targeting approaches, enhancing the quantity and duration of drug circulation in the blood. The antitumor drugs carried by these nano-DDSs can selectively accumulate in tumor tissues, resulting in optimal antitumor effects [28]. Various types of NPs, including polymeric NPs, lipid-based NPs, inorganic NPs, and other nano NPs, have been utilized to form nano-DDSs for OC treatment.

### 3.1. Polymeric NPs

Polymeric NPs are among the most commonly used nanocarriers for drug delivery. They are made from surfactants comprising amphiphilic organic molecules and polymers with low molecular weight [82,83]. Polymeric NPs can effectively encapsulate antitumor drugs, protecting them from degradation in the body. Additionally, their surface can be modified with ligands to achieve targeted delivery to cancer cells [82,84]. By controlling the biodegradation rate of the polymeric NPs and the drug diffusion rate within the matrix, controlled release of the drug can be achieved [84]. Table 2 shows recent studies on polymeric NPs for treating OC.

Polymeric NPs derived from natural polymers like chitosan, proteins, and cassava starch have been applied to form nano-DDSs for OC therapy owing to their drug-loading capability and natural biocompatibility [41,85,86,87,88,89,90,91,92]. Various synthetic polymers, such as polylactic-co-glycolic acid (PLGA) [93], polyethylene glycol (PEG) [64], and polylactic acid (PLA) [94] are also commonly used to form nano-DDSs in OC therapy due to their excellent biocompatibility, biodegradability, and synthetic versatility. Techniques such as nanoprecipitation [64,92,95], cross-linking [86], ionotropic gelation technique [85,88], emulsion solvent evaporation [93,96], and self-assembly [56,89,90] are employed to prepare these nanocarriers. Certain copolymers have been specifically designed to enhance drug-loading capacity and prolong circulating time, making them effective nanocarriers for delivering antitumor drugs in OC therapy [56,66]. Polymeric NPs loaded with chemotherapy drugs can be administered through gels, tablets, films, patches, and injections [9]. These methods offer advantages over traditional oral chemotherapy drugs as they allow for highly selective accumulation and controlled drug delivery in tumors, improved targeting and stability, high biosafety, and extensive drug loading, showing great potential for treating OC [93,97].

**Figure 2:**
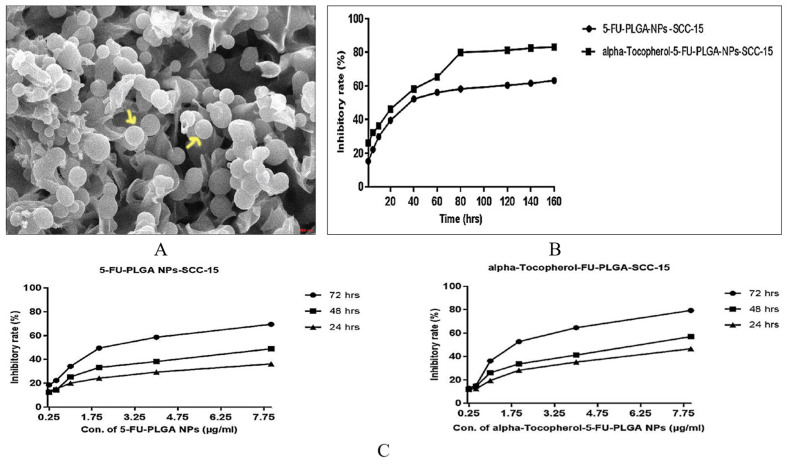
(**A**) Scanning electron microscope of α-t-FU-PLGA/5-FU-PLGA NPs with spherical shape (yellow arrow) and no adherence between the particles. (**B**) Inhibition rate of α-t-FU-PLGA NPs and 5-FU-PLGA NPs on SCC-15 cells over time. (**C**) Inhibition rate of 5-FU-PLGA NPs (left) and α-t-FU-PLGA NPs (right) on SCC-15 cells at different concentrations. Reproduced with permission from ref. [68] (Copyright 2019 Medknow).

PLGA is a biocompatible and biodegradable polymer that can effectively increase the intracellular concentration of antitumor drugs by prolonging blood circulation and escaping the clearance of the mononuclear phagocyte system [30]. In a study by Gupta et al. [95], PLGA was used to nanoencapsulate DTX through the nanoprecipitation method, which resulted in sustained release kinetics. In vitro testing showed that the encapsulated drug had higher anti-proliferation efficiency against SCC-9 cells in a dose-dependent manner compared to free DTX. Additionally, PLGA can be modified with specific ligands for targeted drug delivery to cancer cells. Srivastava et al. [68] developed surface-modified targeted moiety α-tocopherol (α-t) encapsulated with 5-FU-PLGA NPs to treat OSCC. The surface conjugation of α-t on 5-FU-PLGA NPs increased in average particle size ranges from 145 nm to 160 nm. The SEM of the α-t-FU-PLGA/5-FU-PLGA nanoformulation showed spherical shape with easy surface and no adherence between the particles in α-t-FU-PLGA/5-FU-PLGA nanoparticles (Figure 2A). The cellular uptake of α-t-FU-PLGA NPs by SCC-15 cells was higher than that of 5-FU-PLGA NPs. Both α-t-FU-PLGA NPs and 5-FU-PLGA NPs showed sustained inhibition rates on SCC-15 cells for up to 160 h (Figure 2B). In vitro experiments confirmed that α-t-FU-PLGA NPs had a more substantial inhibitory effect on SCC-15 cells than 5-FU-PLGA NPs (Figure 2C).

**Figure 3:**
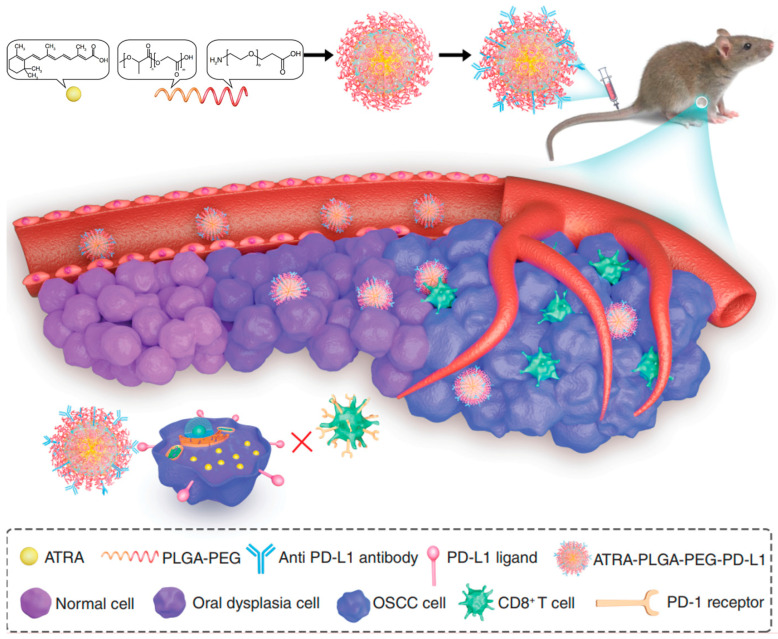
Fabrication of ATRA-PLGA-PEG-PD-L1 nanomedicines. Reproduced with permission from ref. [66] (Copyright 2020 Future Medicine).

Block copolymers prepared from PEG and PLGA have been demonstrated to have prolonged the circulation time [98] and excellent pharmacokinetic parameters [66,99,100]. In a study by Chen et al. [66], nanomedicines were fabricated using all-trans retinoic acid (ATRA) loaded into PLGA-PEG nanocarriers, which were then modified with an anti-PD-L1 antibody for the immuno-therapy of OSCC (Figure 3). The ATRA-PLGA-PEG-PD-L1 NPs effectively inhibited cell proliferation and induced apoptosis in CAL-27 cells, with rapid cellular uptake. In subcutaneous tumor-bearing model mice injected with SCC-7 cells, the AT-RA-PLGA-PEG-PD-L1 NPs targeted the tumor cells more specifically compared to free ATRA, resulting in enhanced anticancer activity and reduced side effects. Furthermore, after treatment, CD8^+^ T cells were activated around PD-L1-positive cells in the TME.

Chitosan, a naturally derived amino polysaccharide with reactive hydroxyl and amino functional groups, can be employed for encapsulating antitumor drugs, with the expectation of exhibiting high permeability of drugs and active substances on mucosal epithelial tissues [101,102]. In a study by Ortega et al. [41], a thermosensitive and mucoadhesive hydrogel was formulated containing chitosan-coated curcumin (Cur)-loaded lipid-core nano-capsules. The results indicated that the hydrogel with chitosan-coated nano-capsules had greater Cur permeation compared to the uncoated formulation.

Among the various NPs under investigation, protein-based NPs such as human serum albumin (HSA) NPs [89] and lactalbumin NPs [91] are emerging as promising bio-inspired nanocarriers due to their natural biocompatibility, non-immunogenicity, high drug loading ability, and tumor-targeting ability. Kumbham et al. [89] developed HSA NPs-mediated tumor-targeted DOX/oleanolic acid (OA) combination therapy (DOX@HSA-OA NPs) and examined its effectiveness in treating OC. The in vitro experiment showed that the NPs were taken up by the cancer cells and demonstrated superior efficacy in killing them compared to monotherapy.

Starch-based NPs are becoming increasingly popular due to their safe nature for human health. A study conducted by Kaokaen et al. [92] revealed that cordycepin (CS)-loaded cassava starch NPs (CC-SNPs) could effectively inhibit the growth of OC cells through the induction of reactive oxygen species (ROS) generation and the reduction in protein secretion, both of which enhanced the activity of CS.

Although polymeric NPs have many advantages as nanocarriers in nano-DDSs for OC therapy, some limitations need to be considered in the design of polymeric NP-based nano-DDSs. Firstly, some polymeric materials may have poor biodegradability, leading to accumulation in the body and potential toxic reactions. Secondly, the load and release rate of the drug may be limited, affecting the effective delivery and release of the drug. In addition, the preparation process of polymeric NPs can be complex, and the cost of large-scale production is high. These limitations can be addressed by selecting polymeric materials with good biodegradability, such as PLA or PEG, to ensure that the DDS can be efficiently metabolized and eliminated. Optimizing the structure and surface modification of polymeric NPs can improve the drug loading and release rate. In addition, studying new preparation processes, finding cost-effective production methods, and considering the feasibility of large-scale production can reduce costs and promote the practical application of polymeric NP-based nano-DDSs in OC therapy.

**Table 2:** Studies on polymeric NPs applied for nano-DDS formation in OC therapy.

Nanocarrier	Active Agent	Assembly Method	Mechanism	Advantages	Ref.
PLGA	Resveratrol NP (Res)	PLGA encapsulated nano form of Res (Res-NP)	Res-NPs obstructed carcinogenesis and metastasis by inhibiting CXCL-12 and IL-6 production in vitro, in vivo, in ovo, and ex vivo systems	Reduced metastasis and angiogenic markers, ovo vascularization, intracellular NO production, matrix metalloproteinase expression, tubular formation, and representative CSCs and angiogenesis markers	[93]
	5-FU	5-FU was conjugated with PLGA by ionic cross-linking, and α-tocopherol used as a functionalized surface moiety (α-t-FU-PLGA NPs)	5-FU-induced apoptotic cell death	Higher apoptosis rate against OSCC and a robust inhibitory effect on SCC-15 cells after 96 h, establishing steady-state inhibition after 160 h incubation compared to 5-FU-PLGA NPs	[68]
	DOX (chemotherapy drug) and indocyanine green (ICG) (PTT agent)	PLGA NPs loaded with ICG and DOX and conjugated with chemokine SDF-1	Anticancer effect of DOX and PTT effect mediated by ICG	Dual-killing effect on OSCC cells and l enhanced cellular uptake and cell apoptosis	[74]
	DTX	PLGA NPs encapsulating DTX	Anticancer effect of DTX	Sustained release of the drug and higher anti-proliferation efficiency against SCC-9 cells	[95]
Poly (β-amino ester) (PBAE)/PLGA blended NPs	ICG (PDT PS and PTT agent)	PBAE/PLGA blended NPs co-loaded with ICG and Nrf2-siRNA, then encapsulated in cancer cell membrane from homologous OTSCC, thus obtaining M@PPI-siRNA	PTT effect mediated by ICG and PDT effect enhanced by Nrf2-siRNA	Reduced tumor cells’ resistance to oxidative stress and amplified anticancer effects of ICG-mediated PDT by maintaining intracellular ROS	[103]
PEG	Aggregation-induced emission photosensitizer 5 (AIEPS5) (PDT PS)	A PEG chain was linked onto AIEPS5, and anti-Her-2 nanobody (NB) was further utilized to achieve targeted delivery of AIEPS5-NPs-NB	Effect of AIEPS5-mediated PDT	Effective ^1^O_2_ generation capability, bright FR/NIR emission centered at 680 nm, and negligible dark toxicity	[72]
	Graphene quantum dots (GQDs) (PDT PS)	GQDs as photosensitizers were conjugated to PEG to enhance solubility and blood circulation	High ROS from GQDs-mediated PDT toxicity killed tumor cells and triggered immune responses by releasing endogenous tumor antigens.	Low cytotoxicity, good solution stability, and strong endocytosis significantly increased host-immunity-related CD8^+^ T cells (cytotoxic T lymphocytes) and pro-inflammatory cytokines IFN-γ and TNF-α	[104]
	PEG-stabilized, PDPN antibody (PDPN Ab)- and DOX ((PDPN Ab)-AuNP-DOX) (Chemotherapy drug and PTT agent)	PEG-stabilized, PDPN antibody (PDPN Ab)- and DOX-conjugated AuNPs	Synergistic anticancer effects for combined chemotherapy and PTT against OC	Low toxicity, high drug-carrying capacity, and cell uptake efficiency, as well as enhanced anti-tumor efficacy combined with laser irradiation	[105]
	DOX (chemotherapy drug) and hematoporphyrin (HP) (PDT PS)	A prodrug of DOX, called RPTD, was synthesized via thioketal linkage and cRGD peptide modification and then used to prepare NPs to encapsulate hematoporphyrin photosensitizer (HP), and HP-loaded RPTD (RPTD/HP) NPs were formulated	Anticancer effect of DOX and PDT effect mediated by HP	Practical effects on inhibiting cell growth and inducing apoptosis, excellent tumor-targeting ability, and significantly inhibited tumor growth	[106]
	DOX	PEGylated DOX (PD) was first synthesized by the conjugation of DOX with bis-amino-terminated PEG via succinyl linkage, and then PD NPs were prepared by a modified nanoprecipitation method. After that, PD NPs were surface-modified with HN-1 to form HNPD NPs	Cytotoxicity of DOX	Higher cellular uptake and cytotoxicity than PD NPs in OSCC cells, significantly enhanced tumor-targeting and penetration efficiency compared with PD NPs, and inhibited tumor growth	[64]
PDA	Black phosphorus nanosheets (BP NSs) (PTT agent)	PDA-modified BP NSs as basal material, polyacrylamide hydrochloride-dimethylmaleic acid (PAH-DMMA) charge reversal system for further surface modification (BP@PDA-PAH-DMMA)	Excellent photothermal properties and tumor enrichment ability of BP@PDA-PAH-DMMA	Enhanced uptake of OC cells, excellent photothermal properties and tumor enrichment ability, and a good killing effect on OC cells	[107]
	BiVO_4_/Fe_3_O_4_@ PDA superparticles (SPs) (PTT agent)	BiVO_4_ and Fe_3_O_4_ NPs were first prepared, followed by their subsequent self-assembly into BiVO_4_/Fe_3_O_4_ SPs via the oil-in-water microemulsion route. After that, the as-prepared BiVO_4_/Fe_3_O_4_ SPs were covered by PDA	Photothermal effect mediated by Fe_3_O_4_ NPs	Improved the photothermal conversion capability, superior synergistic therapeutic efficacy on tumors	[108]
	DOX (chemotherapy drug) and terbium ion-doped hydroxyapatite (HATb) NP (PTT agent)	PDA encapsulated both HATb NP as a luminescent probe and anticancer DOX	HATb–PDA–DOX plus NIR treatment synergistically promoted the overproduction of ROS, cell cycle arrest, and increased cell apoptosis.	PH/NIR responsive release characteristics and a better antitumor effect on OSCC cells than chemotherapy or PTT alone through the overproduction of ROS, cell cycle arrest, and increased apoptosis	[109]
PLA	CDDP and chloroquine (CQ)	PLA combined with CDDP/CQ-PLA NPs and PLA combined with CDDP NPs (CDDP-PLA NPs)	CDDP/CQ-PLA NPs reduced autophagy and enhanced ROS and apoptosis of CAL-27 cells	Good drug loading capacity and drug release, higher ROS and apoptosis rates and lower autophagy	[94]
Polyethylene glycol-polyethyleneimine	Wnt-1 siRNA (gene therapy drug) and Chlorin e6 (Ce6) (PDT PS)	Polyethylene glycol-polyethyleneimine-Ce6 (PEG-PEI-Ce6) NPs loading Wnt-1 small interfering RNA (siRNA)	PEG-PEI-Ce6 NP-mediated PDT inhibited cell growth and enhanced the cancer cell-killing effect	Inhibited Wnt/β-catenin signaling pathway and reduced the expression of Wnt-1, β-catenin, and vimentin, inhibited cell growth and significantly enhanced killing effect on cancer cells.	[110]
Fluorophore-modified poly(L-lysine) (PL-647)	Platinum prodrug	Hybrid nano-architectures (gold ultrasmall NPs linked by PL-647) enclosing a platinum prodrug and decorated with a customized peptide (Nas-cispt-tf2)	Anti-angiogenic and pro-apoptotic effects of platinum prodrug by the downregulation of the vascular endothelial growth factor gene and increased expression of cleaved caspase-3.	Increased accumulation of Nas-cispt-tf2, enhanced tumor volume reduction effect, increased expression of cleaved caspase-3 protein	[63]
γ-polyglutamic acid	Gefitinib (Gef) and Cur	γ-polyglutamic acid-coated NPs loaded with Gef and Cur (γ-PGA-Gef/Cur NPs)	Free Gef/Cur and γ-PGA-Gef/Cur NPs induced apoptotic cell death via caspase- and mitochondria-dependent pathways	Significantly reduced overall viability of SAS cells, significantly inhibited tumor size	[96]
Amphiphilic mPEG-PLA	Tetravalent platinum prodrug Pt (IV)-diazide (chemotherapy drug) and Ce6 (PDT PS)	Nanosize micelles self-assembled by amphiphilic mPEG-PLA, photosensitizer Ce6, and tetravalent platinum prodrug Pt (IV)-diazide	Irradiation activated Ce6 and photodecomposed to produce cytotoxic Pt (II), azidyl radical, and molecular oxygen.	Activated Ce6 upon laser irradiation and photodecomposed to produce cytotoxic Pt (II), azidyl radical, and molecular oxygen, producing a robust antitumor response, revealing its great potential to reverse hypoxia in chemo-photodynamic combination therapy.	[111]
H20-PLA@ PDA-PEG-FA NPs	DOX (chemotherapy drug) and PDA (PTT agent)	DOX-loaded polymeric NPs (DOX/H20-PLA@PDA NPs) were functionalized with amino-poly (ethylene glycol)-FA (H2N-PEG-FA) after coating them with PDA to form the targeting combination, DOX/H20-PLA@PDA-PEG-FA NPs.	Photothermal effect and the pH sensitivity of the PDA films; chemotherapy effect of DOX	Very effective therapeutic effect on OC, accelerated drug release in acidic TME under laser irradiation	[62]
PLGA-PEG nanocarriers	ATRA- PLGA- PEG-programmed death-ligand 1 (PD-L1) nanomedicines	ATRA-PLGA-PEG-PD-L1 nanomedicines were fabricated by loading ATRA into PLGA-PEG nanocarriers and modification using an anti-PD-L1 antibody	ATRA-PLGA-PEG-PD-L1 NPs inhibited proliferation and induced apoptosis in cancer cells	Rapid cellular uptake in DOK and CAL-27 cells, significantly inhibited proliferation and inducing apoptosis, specifically targeted tumor cells, enhanced anticancer activity and reduced side effects	[66]
FA-PEG-TK-PLGA NPs	DOX	PLGA, along with PEG, was used in the significant skeleton, and the ROS-responsive thioketone-containing (TK) was used for FA ligation to form FA-PEG-TK-PLGA NPs to load DOX	DOX-induced apoptotic cell death	Effective escape from endosomes, quick release of the entrapped DOX into the cytoplasm, induced apoptosis of OSCC cells	[56]
Poly (ethylene glycol)-poly (ε-caprolactone) copolymers (PEG-PCL)	ICG (PDT PS) and an organic compound (C3) (PTT agent)	C3 encapsulated in PEG-PCL with ICG to form hybrid NPs (PEGs-PCLs-C3s-ICG NPs)	PEGs-PCLs-C3s-ICG NPs simultaneously produced hyperthermia through C3 and produced ROS with 808snm laser irradiation at tumor sites	Better photothermal conversion stability, lower cytotoxicity, and a faster metabolic rate, which ensured the tumor elimination effect of PTT in vivo	[112]
Alginate hydrogel	CDDP (chemotherapy drug) and AuNPs (PTT agent)	A multifunctional nano platform comprising alginate hydrogel co-loaded with CDDP and AuNPs (abbreviated as ACA)	Tri-modal (thermo-chemo-radio) therapy effect	Induced a superior anticancer efficacy than mono- or bi-modality treatments, morphological features of KB cell injury and apoptosis	[113]
A self-destructive aliphatic polycarbonate	DOX (chemotherapy drug) and Ce6 (PDT PS)	DOX was conjugated to a self-destructive polymeric carrier through a ROS-sensitive pendant thioketal bond (PEG-PBC-TKDOX). Then, Ce6 was loaded through the π−π stacking interaction with DOX.	Anticancer effect of DOX and PDT effect mediated by Ce6	Light stimulated Ce6 to produce cytotoxic ROS and spatiotemporally activated a cascade reaction to release the loaded drugs	[114]
Nano DOX-ICG MMP-responsive hydrogel (NDIMH)	DOX (chemotherapy drug) and ICG (PDT PS)	Nano DOX-ICG MMP-responsive hydrogel (NDIMH)	Anticancer effect of DOX and PTT effect mediated by ICG	Effectively inhibited viability, invasion, and metastasis of SCC-15 cells in vitro, exhibiting favorable synergistic antitumor efficacy and acceptable biosafety, significantly improving the retention of nanodrugs at the tumor site	[115]
Chitosan	Ursolic acid	Ursolic acid encapsulated with chitosan (UACNP)	The anti-lipid peroxidative/antioxidant properties of UACNP during DMBA-mediated oral tumor growth	Significant antitumor effects in the pre-initiation and post-initiation phases in experimental oral carcinogenesis in Syrian golden hamsters	[85]
	LNCc (Cur lipid-core nanocapsules)	Hydrogel containing LNCc coated with chitosan	The formulations presented intrinsic cytotoxic activity	Significant reduction in cell viability of oral squamous cell lines in all test groups	[41]
	miR-144-source of macrophage-derived epolar [MEXO]/CA-miR-451a	Chitosan was prepared using the ionic cross-linking method, and biomimetic NPs coloaded with the miR-144/451a cluster were prepared using the uptake–efflux method	miR-144/451a cluster synergistically inhibited the proliferation, migration, and invasion of OSCC	Substantially reduced viability, migration, and invasion of OSCC. Calcium-binding protein 39 (CAB39) and migration inhibitory factor (MIF) expression in OSCC treated with miR-144-MEXO/CA-miR-451a NPs decreased significantly compared to the miR-144/451a group.	[86]
	GBAS gene plasmid DNA (shGBAS) (gene therapy drug) and 5-aminolevulinic acid (5-ALA) (PDT PS)	5-ALA photosensitizer-loaded chitosan (CS) NPs were prepared using the ionic crosslinking method and further synthesized with the GBAS gene plasmid DNA (shGBAS) by electrostatic attraction (CS-ALA-shGBAS NPs)	Effect of 5-ALA mediated PDT and gene therapy effect of shGBAS	Good dispersion, stability, and hypotoxicity, showing a good mitochondrially targeted killing effect on OSCC in vitro and in vivo	[116]
	Simvastatin (SIM) and Quercetin (QRC)	In situ gel (ISG) loaded with chitosan-coated SIM NPs doped with QRC (chitosan-coated SIM–QRC NPs)	Enhanced biological activity of SIM due to QRC	Slower drug release rate, significantly increased apoptosis mediated by caspase-3 and increased level of tumor suppressor protein, enhanced bioactivity of SIM	[87]
	Phloretin	Phloretin-loaded chitosan NPs (PhCsNPs)	PhCsNPs-mediated tumor cell apoptosis	Enhanced cellular uptake, sustainable release, and bioavailability of phloretin	[88]
Fluorinated chitosan	Ce6 (PDT PS)	Fluorinated chitosan-Ce6 and catalase co-assemble to form stable NPs	Effect of Ce6-mediated PDT and intracellular H_2_O_2_ transformation into O_2_ under catalase catalysis	Significantly improved cross-membrane penetration capacity, better anticancer activity	[117]
PLGA-Chitosan Janus NPs (JNP)	IL-6 receptor antagonist, tocilizumab (TCZ)	TCZ loaded JNP	Antagonistic effect of TCZ on IL-6 and JNP enhanced oral keratinocyte internalization	Good mucosal penetration and significant xenograft inhibition and outperformed all control groups in inhibiting tumor cell proliferation, reducing tumor size, and reducing proto-oncogene ERG expression	[118]
Chitosan and PLGA-based NPs and polylactic acid fibers	18-β-Glycyrrhetic Acid (GA)	GA coated with chitosan and PLGA (GA-NPs) and GA coated with polylactic acid fibers (GA-FBs)	Cytotoxicity of GA on PE/CA-PJ15 cells	Cytotoxic effect on PE/CA-PJ15 cells but had no cytotoxic effects on human gingival fibroblasts, increased sensitivity of cancer cells to ROS over-production	[119]
Catechol-modified chitosan/hyaluronic acid NPs	DOX	Catechol-modified chitosan/hyaluronic acid NPs loading DOX (DOX-NPs)	DOX-NPs were taken up, accumulated, and induced apoptosis in cells	Superior mucoadhesive capability, inhibited growth of HN22 cell lines with low IC_50_, more extensively taken up, accumulated, and induced apoptosis in cells than free DOX	[120]
Anionic protein-Chitosan-Ag_3_AuS_2_ hydrogel	Ag_3_AuS_2_ NPs (PTT agent)	Complexation of genetically engineered anionic protein (E_72_), chitosan, and Ag_3_AuS_2_ NP (termed as E72-Chitosan-Ag_3_AuS_2_)	Photothermal effect mediated by Ag_3_AuS_2_ NPs	Good biocompatibility and ultra-strong photothermal effect with no side effects on surrounding normal tissues and suppressed potential tumor recurrence	[121]
Sucrose esters	Ce6 (PDT PS)	The emulsion-solvent diffusion method was used to prepare the nano sucrose esters encapsulating Ce6	Singlet oxygen generation and cytotoxicity to OSCC cells induced by Ce6-mediated PDT	Higher drug encapsulation efficiency and a faster drug release rate than pure Ce6, promoted cell uptake of Ce6, singlet oxygen production in vitro, and cytotoxicity to OSCC cells	[122]
Polymeric nanoemulsions	Methylene blue (MB) (PDT PS)	Polymeric nanoemulsions containing MB	Effect of MB-mediated PDT	Effectively reduced cell viability, a significant decline in viability for all fluences used, enhanced internalization of the adopted biological model, further reducing toxicity and reducing adverse reactions	[123]
Fucoidan (FU)/hyaluronic acid (HA) cross-linked zein (Zn) NPs	Fisetin (FS)	FS-loaded cross-linked Zn NPs (ZFH), which contained HA& FU	Significant cytotoxic action of ZFH and enhanced uptake of ZFH by tumor cells	Remarkable uptake by SCC-4 cells with a significant cytotoxic effect, significantly decreased OSCC-specific serum biomarkers and histological tumor grades, and increased caspase-3 levels	[69]
Hyaluronic acid (HA)	CDDP (chemotherapy drug) and TQTPA [4,4′-((6,7-bis(4-(hexyloxy)phenyl)-[1,2,5]thiadiazolo [3,4-g]quinoxaline-4,9-diyl)bis(thiophene-5,2-diyl))bis(N,N-diphenylaniline)] (PTT agent)	Multimodal NPs (NPs) loading TQTPA and CDDP (HT@CDDP) by hyaluronic acid.	Combined PTT effect with chemotherapy	Good tissue penetration quality and active targeting ability, outlined orthotopic tongue tumors and metastatic lymph nodes as small as 1 mm in nude mice by IR-808 under NIR exposure, biocompatibility and low systematic toxicity	[124]
A drug-mimicking peptide hydrogel named L-NILMDP	Cyclic dinucleotide (CDN)	L-NILMDP was loaded with immunotherapy agonist CDN	The combination of the L-NIL-MDP hydrogel with its inherent inducible nitric oxide synthase (iNOS) inhibition and the controlled release of stimulator of interferon genes (STING) agonist immunotherapy	4- and 20-fold slower drug release rates than commercially available hydrogels, allowing the immune-mediated elimination of established treatment-resistant oral tumors with longer median survival than the untreated group	[125]
Cathepsin B-responsible NPs linear-dendritic mPEG5000-BMA4	AKT inhibitor capivasertib (AZD5363)	Linear-dendritic mPEG5000-BMA4 encapsulated capivasertib	Capivasertib was a potent agent that sensitized radioresistant OSCC cells to irradiation (IR)	Greatly enhanced tumor cell suppression in 3D cell cultures and OSCC tumor shrinkage compared to IR alone	[126]
Oleanolic acid-conjugated human serum albumin NPs	DOX	HAS and oleanolic acid were conjugated to form self-assembled NPs that entrapped DOX (DOX@HSA-OA)	DOX@HSA-OA NPs-mediated DOX penetration, DNA damage, oxidative stress, and apoptosis-induction	Lower IC_50_ value than DOX against FaDuHTB-43 at various time points, higher apoptosis and cell cycle arrest (G2/M phase); the DOX@HSA-OA NPs-mediated DOX penetration and cell death/shrinkage were significant in FaDu-HTB-43 spheroids.	[89]
Human serum albumin-poly (Lactide)-conjugated self-assembly NPs	DTX	The DTX-loaded DTX@HSA-(PLA)_2_ NPs were prepared by the desolvation-self-assembly technique	The DTX@ HSA-(PLA)_2_ NPs treatment induced apoptotic marker expressions, cell-cycle arrest in the G2/M-phase, DNA damage, and mitochondrial depolarization	Greater penetration, resulting in the highest cytotoxic response in cancer cells grown in monolayers or spheroids compared to free DTX and DTX-loaded in HSA NPs, improved half-life of DTX, plasma residence time, and decreased clearance than free DTX.	[90]
HSA	CDDP (chemotherapy drug) and ICG (PDT PS and PTT agent)	Human serum albumin carrying ICG-CDDP NPs (HSA-ICG-CDDP NPs)	Anticancer effect of DDP and PDT and PTT effect mediated by Photosan-2	Precisely being triggered to release DDP under NIR irradiation at 808 nm, more potent antitumor effects than the treatment with ICG, HSA-ICG, and DDP alone	[127]
Lactalbumin NPs	Genistein nano-formulation	Genistein-loaded lactalbumin NPs (GLNPs) were prepared by using the antisolvent precipitation method	GLNPs withdrew epigenetic transcriptional repression and selectively induced apoptosis in human OSCC	Selectively induced OSCC cell apoptosis compared with normal fibroblasts, causing the withdrawal of epigenetic transcription repression by simultaneously down-regulating polycomb group protein (PcG) and its subsequent targets, regulating EZH2 expression through proteasomal-mediated degradation and 3PK inhibition	[91]
Nano-cassava starch	Cordycepin (CS)	Cordycepin-loaded cassava starch NPs (CCSNPs)	CS had anti-oxidant properties capable of inhibiting the growth and promoting the death of cancer cells	Increased HSG proliferation, protein secretion, and salivary-specific gene, AMY, and AQP5 expression, protecting and clearing ROS by stimulating antioxidant genes in HSGs, inhibiting the growth of HSC-4 cells by stimulating ROS generation and reducing protein secretion	[92]

### 3.2. Lipid-Based NPs (LBNPs)

**Figure 4:**
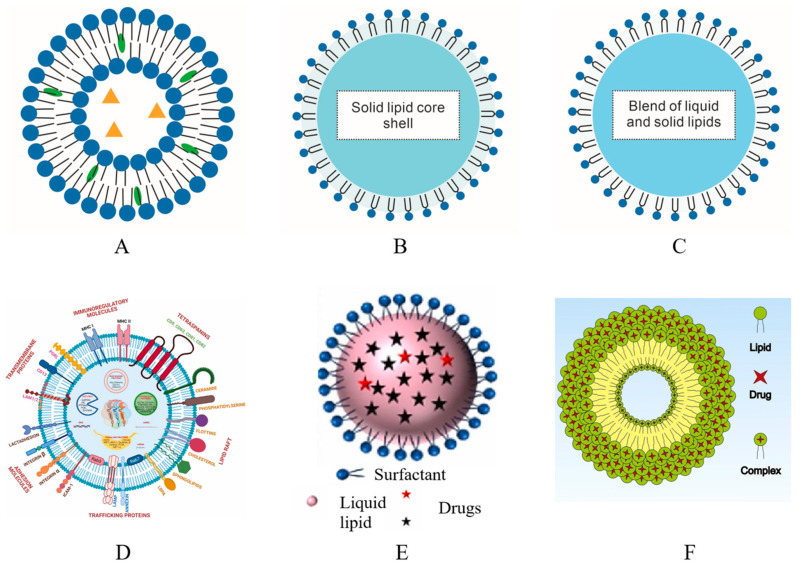
Various types of LBNPs. (**A**) Liposomes: liposomes can encapsulate hydrophobic drugs (green ovals) in the hydrophobic region and hydrophilic ones (orange triangles) in the interior aqueous region. (**B**) SLNs consisting of matrix materials and surface stabilizers. (**C**) NLCs composed of solid lipids, liquid lipids, and surfactants. (**D**) Elemental composition of salivary exosomes. Reproduced with permission from ref. [153] (Copyright 2022 Elsevier). (**E**) Elemental composition of NEs. Reproduced with permission from ref. [158] (Copyright 2023 Elsevier). (**F**) Elemental composition of PLC-NPs. Reproduced with permission from ref. [160] (Copyright 2013 Elsevier).

LBNPs refer to an NP system composed of lipids, including fatty acids, fats, and phospholipids [128]. Some LBNPs are expected to enhance drug bioavailability, promote drug accumulation in tumor tissues, and increase drug circulation time [129]. Depending on the lipid component used and the preparation method, various types of LBNPs are used to treat OC, including liposomes [130,131], solid lipid NPs (SLNs) [58,71], nanostructured lipid carriers (NLCs) [132,133], exosomes [134,135], lipid nanoemulsions (NEs) [136], and phospholipid complex loaded NPs (PLC-NPs) [137] (Figure 4). Recent studies on the use of LBNPs for nano-DDS formation in treating OC are summarized in Table 3.

Liposomes are spherical structures composed of enclosed phospholipid bilayers, which can carry hydrophilic drugs in their aqueous core and lipophilic drugs in their lipid bilayer [138,139,140] (Figure 4A). Due to their high biocompatibility, bioactivity, stability, and flexibility, liposomes are the most frequently employed LBNPs in cancer therapy [141]. Jin et al. [142] developed methotrexate (MTX)-loaded liposomes using the thin film hydration method and cast in an optimized mucoadhesive film to establish a mucoadhesive patch for treating OC. The MTT assay showed a significant decrease in the half-maximal inhibitory concentration of MTX in HSC-3 cells with the MTX-loaded liposomes. Additionally, the apoptosis rate in HSC-3 cells was almost tripled with MTX-loaded liposomes.

SLNs and NLCs have gained popularity in recent years as effective carriers for cancer therapy due to their ability to increase drug stability, improve entrapment efficiency for hydrophobic molecules, and enhance oral drug absorption [143,144]. SLNs are colloidal particles that remain solid at both room and body temperature. They consist of matrix materials such as triglycerides, fatty acids, cholesterol, waxes, partial glycerides, fats, and surface stabilizers like phospholipids, bile salts, soybean lecithin, egg lecithin, poloxamers, and polysorbates. The solid lipid core is enclosed in a lipid monolayer, and hydrophobic drugs can be encapsulated within the central solid-lipid core of SLNs with the presence of suitable surfactants [139,143,144] (Figure 4B). A novel approach to treating OSCC was developed by Bharadwaj and Medhi [58], who fabricated FA-conjugated SLNs loaded with PTX and ascorbic acid (AA). The FA-conjugated SLNs showed a biphasic drug release behavior both in vitro and in vivo. A higher efficiency was observed when FA-conjugated PTX-loaded SLN and FA-conjugated AA-loaded SLN were combined as compared to when used individually in vivo.

NLCs are a newer type of lipid carrier first developed to overcome the limitations of SLN loading efficiency [145]. They are composed of a combination of solid and liquid lipids, along with surfactants (Figure 4C). This unique composition allows NLCs to effectively encapsulate both lipophilic and hydrophilic drugs and prevent drug leaching and oxidation during storage, making them an excellent option for drug delivery [139,146]. Furthermore, NLCs can be modified on their surface to control drug delivery, improve the solubility of hydrophobic drugs, and achieve specific targeting of drugs [147,148]. In a study by Shete et al. [133], an NLC-loaded metformin hydrochloride (MET) delivery system was developed to improve the hydrophilicity of the drug. The optimized nano-DDS demonstrated a high MET release rate within 24 h. It also exhibited significant cytotoxicity against KB OC cells, with smaller IC_50_ values compared to the MET solution in vitro.

Exosomes are cell-derived extracellular vesicles (EVs) produced through endosomal networks and released into the extracellular space through the exocytosis of multi-vesicular bodies [149,150,151]. They are enclosed by a phospholipid bilayer and safeguard and convey various cargoes such as nucleic acids, diverse forms of proteins, and a unique range of lipids [152,153] (Figure 4D). Exosomes can be secreted by different types of cells, including tumor cells, and are typically 30–100 nanometers in size [154]. Their unique structure and composition make them valuable for a range of applications. During the initiation and growth of tumors, various cellular functions such as down-regulation of tumor suppressors, enhancement of cell proliferation and invasion, initiation of angiogenesis, and metastasis require exosomes for intercellular communication [155,156,157]. In OC therapy, specific types of cells like dendritic cells, natural killer cells, and mesenchymal stem cells secrete exosomes with special properties and therapeutic potential that could be used in various aspects of OC treatment [153]. For example, immune cells, including dendritic cells and other antigen-presenting cells, can secrete exosomes that promote the immune response of target cells by carrying specific drugs or miRNAs and other substances. Genetically engineered exosomes loaded with specific miRNAs, siRNAs, or other therapeutic molecules can be utilized for exosome-based gene therapy. Additionally, exosomes derived from mesenchymal stem cells can significantly impact the function of immune cells and have potential immunomodulatory effects [153]. Furthermore, exosomes are also promising for drug delivery in OC therapy, as therapeutic agents delivered by exosomes can reduce the malignancy of cancer cells. Exosomes can be obtained through ultracentrifugation [134,135], and therapeutic agents can be loaded onto exosomes through co-incubation [134] and electroporation [135].

In a study conducted by Deng et al. [134], the inhibitory effect of miR-34a on OSCC cell proliferation, migration, and invasion was investigated. HN6 OSCC cells were co-incubated with miR-34a-loaded exosomes, resulting in a significant reduction in cell proliferation, migration, and invasion as well as inhibition of SATB2 expression. Another study by Kase et al. [135] utilized exosomes derived from normal fibroblast transfected with Epstein-Barr Virus Induced-3 (EBI3) cDNA and lymphocyte cytoplasmic protein 1 (LCP1) siRNA, referred to as octExosomes, to evaluate their specificity, effectiveness, and antitumor activity. The results showed that octExosomes stably and efficiently transferred siLCP1 into OSCC cells, leading to a downregulation of LCP1 expression compared with their counterparts and ultimately exhibiting a significant tumor-suppressor effect in vitro and in vivo.

Lipid NEs are a type of drug delivery vehicle made of liquid lipids instead of solid ones (Figure 4E). They offer several advantages, including long-term stability, easy preparation, and the effective solubilization of drug molecules [136,158]. Liu et al. [136] discovered that loading Cur onto lipid NEs significantly improved its bioavailability. Additionally, Cur-NEs demonstrated a significant dose-dependent cytotoxic effect on OSCC cells compared to the control.

Different from liposomes encapsulating hydrophilic drugs in their aqueous core and lipophilic drugs in their lipid bilayer, PLC-NPs are relatively stable complexes formed by charge transfer between drugs and phospholipid molecules, which can change the physical and chemical properties of the carried drug and improve bioavailability, and their preparation method is simple [137,159,160] (Figure 4F). Liu et al. [137] evaluated the sustained drug release effect of free salvianolic acid B (SAB) versus SAB phospholipid complex loaded NPs against oral carcinogenesis in 4NQO-induced oral carcinogenesis-bearing model mice. The results showed that nano-SAB had better chemopreventive effects by promoting anti-proliferation and cell cycle arrest responses.

Lipid-based NPs may have limitations in biodegradability, targeting performance, drug-loading capacity, and stability as a nanocarrier of nano-DDSs in OC therapy. To overcome these limitations, the selection of lipid-based materials with high biodegradability, the introduction of targeting ligands, the combination with other delivery systems, and appropriate packaging or modification can be taken into consideration to improve drug delivery and therapeutic efficacy.

**Table 3:** Studies on LBNPs applied for nano-DDS formation in OC therapy.

Nanocarrier	Active Agent	Assembly Method	Mechanism	Advantages	Ref.
Liposomes	Evodiamine (EVO) (chemotherapy drug) and ICG (PDT PS)	EI@Lipo (theragnostic nanoliposome system) encapsulated EVO, and ICG was fabricated using a typical thin-film spin evaporation process.	Chemo-antitumor effect and PDT effect of EI@Lipo encapsulating EVO and ICG and peroxidase-like catalytic activity of EVO	Theragnostic liposomes had a significant inhibitory effect on in situ tongue tumors by photodynamic combined chemotherapy.	[130]
	DOX	Liposome-coated DOX and Doxil	Apoptotic effect on tumor cells of DOX and Doxil	Compared with DOX, Doxil had a higher apoptosis effect on CAL-27 cells, a higher elevation of caspase-3 levels, and a higher inhibition rate of C-Myc mRNA.	[131]
	CDDP (chemotherapy drug) and Photosan-2 (PDT PS)	Encapsulating CDDP into liposomes to form lipid-platinum-chloride NPs (LPC NPs)	Anticancer effect of CDDP and PDT effect mediated by Photosan-2	PDT+LPC significantly reduced tumor volume. PDT+LPC or LPC treatment showed minimal side effects on renal damage compared to CDDP or the PDT+CDDP group. PDT+LPC prolonged tumor growth inhibition, thereby reducing the dose of chemotherapy drugs.	[161]
	DOX	Hybrid alginate/liposomes systems loading DOX	Cytotoxic effect on tumor cells of DOX	Alginate paste incorporating DOX-loaded liposomes presented similar release rates and was highly influential in promoting cancer cell death.	[162]
	MTX	MTX-loaded liposomes were prepared using the thin film hydration method. These liposomes were cast in the optimized mucoadhesive film to form MTX-entrapped liposomal film (M-LP-F7)	M-LP-F7 exerted a pro-oxidant effect in HSC-3 cells	Caused a significant decrease in the half-maximal inhibitory concentration of MTX on HSC-3 cells. Increased the apoptosis rate in HSC-3 cells by almost 3-fold.	[142]
SLNs	HuR (ELAVL1, an RNA-binding protein) CRISPR and epirubicin	SLNs modified with pH-sensitive epidermal growth factor receptor (EGFR)-targeting and nucleus-directed peptides carrying HuR CRISPR and epirubicin	CRISPR/Cas9 suppressed proliferation, metastasis, and resistance in SAS cells. The cotreatment of epirubicin and HuR CRISPR in SAS cells further facilitated apoptosis/necroptosis/autophagy and caused cancer cell death	CRISPR/Cas9 successfully knocked out HuR and inhibited SAS cell proliferation, metastasis, and drug resistance. Epirubicin and HuR CRISPR worked together to further promote apoptosis/necrosis/autophagy of SAS cells, resulting in cancer cell death. Combined with HuR CRISPR NPs, epirubicin NPs’ anticancer effect and safety significantly improved in SAS tumor-bearing mice.	[71]
	PTX and AA	FA-conjugated SLN loaded with PTX and AA	Cytotoxic effect of PTX and AA	Showed a biphasic drug release behavior. Had a higher efficiency when FA-conjugated PTX-loaded SLN and FA-conjugated AA-loaded SLN were combined compared to when used individually in vivo.	[58]
	ATRA	Phosphatidylethanolamine polyethylene glycol (PE–PEG) coated SLN loading ATRA	Chemotoxic effect of ATRA	The presence of PE–PEG improved active cell internalization of the NPs in SCC-25 cells and reduced the non-specific internalization mechanism. Delivery of ATRA into PE–PEG-coated SLNs increased their chemotoxic effect compared to non-coated SLNs.	[163]
	PTX, 5-FU, and AA	PTX, 5-FU, and AA entrapped SLNs	Cytotoxic effect on tumor cells of PTX, 5-FU, and AA	SLNs exhibited a biphasic nature of drug release both in vitro and in vivo. SLN loaded with PTX and SLN loaded with AA showed greater efficacy in the in vivo treatment of OSCC.	[164]
NLCs	Pitavastatin (PV) and *Pinus densiflora* (Pd) oil	NLCs containing PV combined with Pd oil	Cytotoxicity of PV and Pd oil against HGF-1	Had reasonable dissolution efficiency, good rheological properties, and vigorous cytotoxic activity against the HGF-1 cell line.	[132]
	MET	MET encapsulated with NLC	Significant toxicity of _GMS_MET-NLCs in KB cells and an excellent inducing rise in ROS levels involved in ROS-mediated KB cell death	Achieved a MET release rate of up to 88% in 24 h. Showed significant cytotoxicity to KB OC cells with reduced IC_50_ values compared with the MET solution. Showed a substantial increase in intracellular ROS levels.	[133]
	Silymarin (SME)	SME was loaded in NLCs and further incorporated in mucoadhesive in situ gel (SME-NLCs-Plx/CP-ISG)	ROS generation potential and SME-NLCs-Plx/CP-ISG induced apoptosis at Sub-G0 phase owing to higher penetration of SME-NLCs	A sustained release effect for SMEs indicated enhanced oral mucosa retention. The IC_50_ value was significantly lower than that of SME-NLCs and plain SME. Had a higher inhibitory effect on human KB OC cells.	[165]
	QRC and piperine	QRC and piperine-enriched NLCs	Cell cycle arrest effect of QRC and piperine	Dual drug-loaded NLCs were more effective than the pure drug solution. Improved apoptosis in NLCs. Efficient distribution in various parts of the oral cavity through oral administration.	[166]
HEK293T cell exosomes	MicroRNA-34a	Cholesterol-modified microRNA-34a loaded into HEK293T cell exosomes by co-incubation	MicroRNA-34a-loaded exosomes led to significant inhibition of HN6 cell proliferation, migration, and invasion by down-regulating SATB2 expression	Absorbed by HN6 oral squamous carcinoma cells and significantly inhibited the proliferation, migration, and invasion of HN6 cells by down-regulating SATB2 expression.	[134]
Mesenchymal stem cell-derived exosomes	MiR-155 inhibitor	MiR-155 inhibitor-laded exosomes	MiR-155 inhibitor resulted in the upregulation of FOXO3a (Forkhead box O3-, a direct target of miRNA-155) and induction of the mesenchymal-to-epithelial transition with improved sensitization to CDDP	Pinned down the stem-cell-like behavior, reversed the epithelial-to-mesenchymal transition process, and enhanced drug sensitivity through up-regulation of FOXO3a in drug-resistant xenograft OC models.	[167]
Exosomes from normal fibroblasts transfected with Epstein–Barr Virus Induced-3 (EBI3) cDNA	siRNA of lymphocyte cytoplasmic protein 1 (LCP1)	Exosomes from normal fibroblasts transfected with Epstein–Barr Virus Induced-3 (EBI3) cDNA were electroporated with siRNA of lymphocyte cytoplasmic protein 1 (LCP1) as octExosomes	The silencing of LCP1 by siRNA suppressed both cancer cell growth and metastatic phenotypes	Able to transfer siLCP1 stably and efficiently into OSCC cells, LCP1 was downregulated in OSCC cells with octExosomes compared to their counterparts, thus having a significant tumor-suppressor effect in vitro and in vivo.	[135]
Exosomes derived from microRNA-101-3p-overexpressing human bone marrow mesenchymal stem cells (hBMSCs)	miR-101-3p	HBMSCs-derived exosomes loaded with miR-101-3p	HBMSCs-exosomes combined with miR-101-3p had an excellent therapeutic effect on OC by regulating collagen type X alpha one chain (COL10A1)	Inhibited OC progression. Tumorigenicity assay in nude mice confirmed the inhibitory effects of hBMSCs-derived exosomes, loaded with miR-101-3p, on OC.	[168]
Milk-exosome	DOX (chemotherapy drug) and Ce6 (PDT PS)	Milk-exosomes were conjugated to DOX by a pH-cleavable bond, anthracene endoperoxide derivative (EPT1) and Ce6 were also loaded (Exo@Dox–EPT1 NPs)	Chemo-antitumor effect of DOX and PDT effect mediated by Exo@Dox–EPT1 NPs	When the NPs accumulated at the tumor site, Ce6 produced plasmonic heat and accelerated ROS generation from EPT1 under NIR irradiation. Had synergistic effects of photochemistry, which could be triggered by acid TME and NIR.	[169]
NE	Cur	The Cur-NE formulation was prepared according to the interfacial prepolymer deposition and spontaneous nano-emulsification method	PI3K/Akt/mTOR suppression and miR-199a upregulation mediated by Cur-Nes	Had significant dose-dependent cytotoxicity on HSC-3 cells. Down-regulated PI3K/Akt/mTOR protein expression and up-regulated PI3K-targeting miR-199a expression in a dose- or time-dependent manner. Effectively counteracted the effects of miR-199a inhibitors on OSCC cell proliferation and cell cycle proliferation phase in a time-dependent manner.	[136]
PLC-NPs	SAB	SAB phospholipid complex loaded NPs (nano-SAB)	The blockade of Ki-67, PCNA, and cyclin D1 expression by nano-SAB	Compared with the free-SAB-treated group and 4NQO-exposed group, nano-SAB treatment could effectively inhibit the expression of Ki-67, proliferating cell nuclear antigen (PCNA), and cyclin D1 in high-risk dysplastic lesions. After four weeks of discontinuation, nano-SAB maintained low Ki-67, PCNA, and cyclin D1 expression levels.	[137]

### 3.3. Inorganic NPs

**Figure 5:**
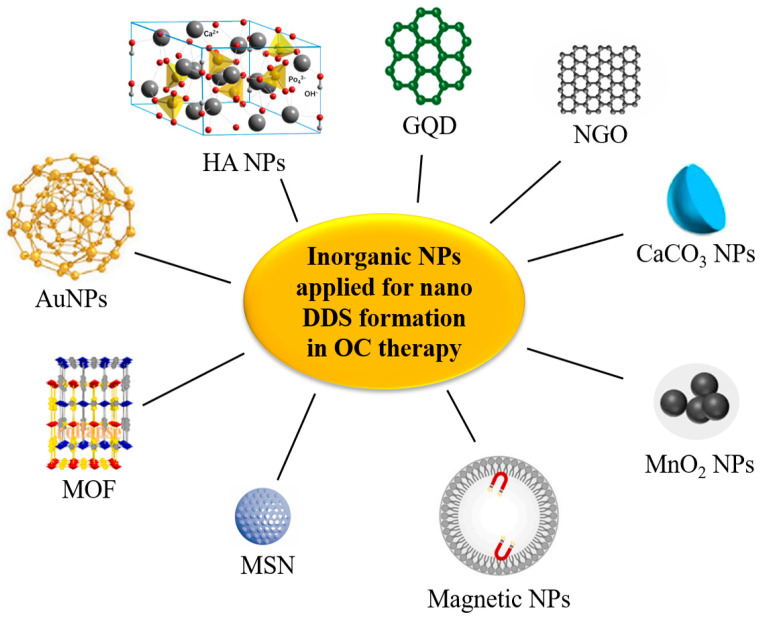
Inorganic NPs applied for nano-DDS formation in OC therapy. Reproduced with permission from ref. [185] (opyright 2023 Elsevier), ref. [42] (Copyright 2023 Elsevier), ref. [186] (Copyright 2023 Elsevier), ref. [21] (Copyright 2019 Elsevier), and ref. [102] (Copyright 2023 Elsevier).

Recent research has demonstrated the potential of inorganic NPs, including Gold NPs (AuNPs) [67,170,171], metal-organic framework (MOF) [60,73,172,173,174,175], mesoporous silica NPs (MSNs) [176,177,178], magnetic NPs [61,179], MnO_2_ NPs [180], CaCO_3_ NPs [181], nanoscale graphene oxide (NGO) [65], graphene quantum dots (GQDs) [182], and hydroxyapatite (HA) NPs [183] as drug delivery vehicles to deliver antitumor drugs in OC therapy (Figure 5). Inorganic NPs possess unique features, such as a large surface area, small volume, ease of synthesis, molecular selective targeting, and a large area-to-volume ratio [5]. Some inorganic NPs have been used to form nano-DDSs for OC therapy, due to their low toxicity, high tolerance, and improved bioavailability as compared to free drugs. Table 4 shows recent studies on inorganic NP applied for nano-DDS formation in treating OC.

AuNPs possess exceptional physical and chemical properties, making them easy to modify with biomolecules, have precise targeting and drug delivery capacity, and exhibit low toxicity [184,185]. Based on these properties of AUNPs, Khamaikawin and Locharoenrat [170] combined DTX-CDDP-FU with AuNPs and applied it to the human KB OC cell line. The resulting nano-DDS demonstrated more significant cytotoxicity against KB cells compared to plain DTX-CDDP-FU, with a lower IC_50_ value. The use of DTX-CDDP-FU with AuNPs could enable sustained release of DTX, CDDP, and 5-FU without compromising their therapeutic effect.

**Figure 6:**
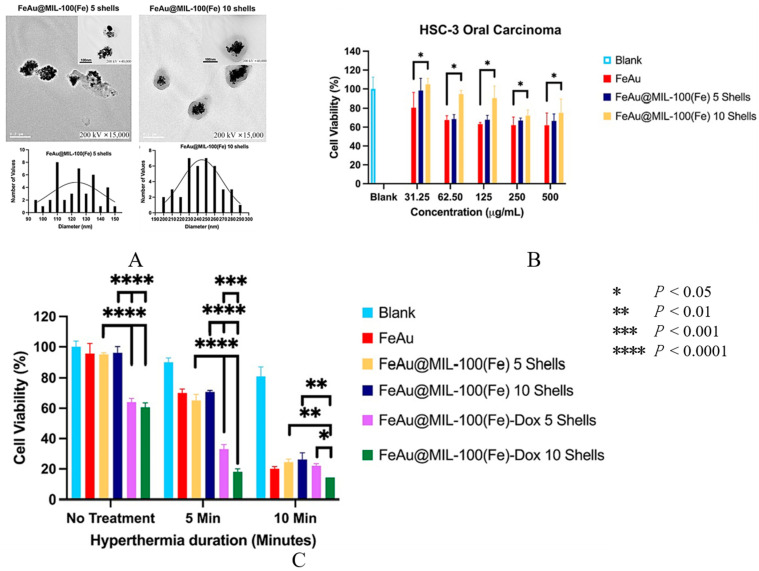
(**A**) TEM micrographs and size distribution of FeAu@MIL-100(Fe) 5-shell nanostructures and FeAu@MIL-100(Fe) 10-shell nanostructures. (**B**) Cell viability of HSC-3 OSCC cells in the presence of FeAu NPs and FeAu@MOF nanostructures. (**C**) Post-hyperthermia cell viability analysis of HSC-3 OSCC cells with or without DOX-encapsulated within FeAu@MOF nanostructures. Reproduced with permission from ref. [173] (Copyright 2022 Elsevier).

MOF materials can be designed to have high drug-loading efficiency and low toxicity [186]. Dhawan et al. [173] reported the production of the FeAu alloy@MOF as a drug delivery platform to limit the growth of OC by generating magnetic hyperthermia and encapsulating DOX in the nanostructures (Figure 6). The study showed that the nanostructures exhibited superparamagnetic and magnetic hyperthermia behaviors. In vitro studies demonstrated that the hyperthermia induced by the nanostructures caused 90% death of HSC-3 cells. In in vivo mice models, the nanostructures reduced the tumor size by 30-fold and tumor weight by 10-fold.

Among the nanocarriers, MSNs show great promise for DDS formation in the treatment of OC. Their tunable pore structure, surface functionalization, and excellent cell internalization allow for maximum loading of small molecules and oligos [177,187,188]. Huang et al. [176] prepared MSNs loaded with 5-FU (MSN-5-FU), collected the outer membrane vesicles (OMVs) of *Escherichia coli* to wrap MSN-5-FU, and then prepared OMVs-MSN-5-FU to explore its effect on lymph node metastasis from OSCC. The results showed that the OMVs-MSN-5-FU DDS could slow down the drug release rate, significantly inhibit OSCC cell proliferation, and control cancer cell metastasis to cervical lymph nodes.

There are still some limitations and corresponding solutions using inorganic NPs as nanocarriers for nano-DDSs in OC therapy. Firstly, these NPs may exhibit potential toxicity. One solution is to choose non-toxic or low-toxic materials and reduce their toxicity through surface modification. Secondly, tumor targeting is limited. This problem can be solved by introducing targeting ligands. Additionally, inorganic NPs have poor biodegradability, which can be enhanced through functional modification. Lastly, the drug loading capacity and release rate may not be ideal, but these can be optimized by adjusting the shape, size, and surface properties of the NPs. By addressing these limitations, the efficacy and safety of OC therapy can be improved using inorganic NP-based nano-DDSs.

**Table 4:** Studies on inorganic NPs applied for nano-DDS formation in OC therapy.

Nanocarrier	Active Agent	Assembly Method	Mechanism	Advantages	Ref.
AuNPs	A triple chemotherapy drug of DTX-CDDP-FU	Au NPs as a vehicle for the delivery of a triple chemotherapy drug of DTX-cisplatin-FU	Cytotoxicity of DTX-CDDP-FU	High loading efficacy of DTX, CDDP, and 5-FU, and had a controllable drug release profile at 24 h. High cytotoxicity on KB cells with lower half-maximal inhibitory concentrations than plain DTX-CDDP-FU.	[170]
	PD-L1 specific antibodies (PD-L1-GNP)	PD-L1-specific antibodies conjugated to AuNPs (PD-L1-GNP)	PD-L1-GNP treatment induced SCC-25 cell apoptosis by inhibiting the function of the PD-L1 protein	Effectively inhibited the growth of SCC-25 cells but did not affect human immortalized keratinocytes (HaCaT). PD-L1-GNP-treated SCC-25 cells showed a phenotype with increased apoptotic proteins.	[67]
	DOX	Two nano-constructs conjugated to DOX through pH-sensitive and pH-resistant linkers (EDC/NHS coupling agents)	Cytotoxic effect of DOX. AuNPs enhanced the cytotoxic effect against cancer cells, inducing a significant cellular apoptosis	PH-resistant DOX NPs were more localized in the nuclei, inducing a 2-fold increase in the apoptotic effect compared with that of pH-sensitive DOX NPs. Higher tumor shrinkage and survival rates in animals treated with DOX pH-resistant AuNPs compared with pH-sensitive ones.	[171]
	Bilirubin	Folate-coated, bilirubin-conjugated gold (FGB) NPs	FGB nanoconjugate suppressed tumor growth in drug-resistant tumor cells by inducing apoptotic cell death	A more substantial inhibitory effect on the activity of multidrug-resistant KB-Ch^R^-8-5 cells. The degree of ROS formation, DNA strand breakage, and apoptosis-induced morphological changes in P-gp-overexpressing drug-resistant cells induced by nanoconjugates were more significant than those induced by bilirubin alone. Had a more substantial inhibitory effect on tumor development in the KB-Ch^R^-8-5 xenograft mouse model than bilirubin treatment alone.	[59]
Gold hybrid NP	Quinacrine (QC)	PLGA-capped hybrid NPs containing QC and Au were formulated by using the oil/water single emulsion solvent evaporation technique (QAuNP)	QAuNP induced the DNA damage and re-replication stress and simultaneously down-regulated the critical repair and replication-related proteins	Excellent anti-CSC growth potential against SCC-9 CSCs and down-regulated representative CSC markers. Prolonged exposure to QAuNP induced S-phase arrest of SCC-9-CSC-like cells and prolonged the G2/M population, leading to cell re-replication and apoptosis. Resulted in the loss of DNA repair in CSCs. Led to excessive DNA damage and re-replication mediated replication stress and replication fork stalling.	[189]
FeAu bimetallic NPs (FeAu NPs)	MMP-1 antibody	Antibodies specific for MMP-1 conjugated to FeAu NPs	Synergistic effect of biomarker-specific antibodies and magnetic NP-induced hyperthermia	A 3.07-fold increase in uptake in HSC-3 cells compared to L929 (fibroblast) cells, which caused a 5-fold decrease in cell viability.	[70]
Zeolitic imidazolate framework-8 (ZIF-8)	USP30 inhibitor MF-094	USP30 inhibitor MF-094 loaded in ZIF-8-PDA and polyethylene glycol-thioketal (PEGTK) to fabricate ZIF-8-PDA-PEGTK NPs	Targeting inhibition of USP30 by MF-094@NPs	USP30 regulated cell viability, glutamine consumption, and apoptosis of OSCC cells by regulating c-Myc deubiquitination. Targeting USP30 through the nano delivery system significantly increased its antitumor effect.	[172]
FeAu alloy@MOF nanostructures	DOX	DOX encapsulated with FeAu alloy@MOF nanostructures	AMF-induced hyperthermia and treatment effect of DOX	Superparamagnetic and magnetic hyperthermia behaviors and caused 90% death of HSC-3 cells, reducing the tumor size by 30-fold and tumor weight by 10-fold.	[173]
Metal-organic framework (IRMOF3)-Zn^2+^	Disulfiram (DSF)	DSF incorporated in the metal-organic framework (IRMOF3)-Zn^2+^, FA) was subsequently loaded on the surface yielding IRMOF3 (IRMOF3-DSF-FA) for targeted therapy of tumors	DSF exerted an antitumor effect via targeted inhibition of ALDH1+ CSCs	High loading capacity, good biocompatibility, and strong cell uptake ability, providing metal ions, target tumor tissues, and inhibit ALDH1+ CSCs, significantly inhibiting CSCs and tumor growth without significant damage to vital organs during treatment.	[60]
Graphene Oxide (GO) @AlFu (aluminum fumarate) MOF	Saponins	Saponin-loaded nanostructure by modifying GO/reduced GO (rGO) with AlFu as MOF core–shell nanocomposite.	The anticancer effect of saponins on altering cell cycle proteins	The survival rate of OSCC cells treated with AlFu–GO–saponin was much lower than that of PDL cells, and the apoptosis of cells treated with AlFu–GO–saponin and AlFu–rGO–saponin was more than that of the untreated group.	[174]
PEGylated nano-graphene oxide	DOX (chemotherapy drug) and NGO (PTT agent)	PEGylated NGO linked DOX and fluorescently-labeled, FAP-targeted peptide chains via hydrogen bonding and π–π bonding.	The thermogenic effect of NPF@DOX simultaneously promoted the local release of DOX and apoptosis based on a pH-stimulated effect.	Exhibiting a high photothermal conversion efficiency under NIR radiation. FAP-targeted NPF@DOX in combination with PTT demonstrated better tumor suppression performance than either therapy alone.	[76]
ZIF-8 nanostructures composed of Zn(NO_3_)_2_·6H_2_O and 2-methylimidazole	DOX	MOFs coated with dental pulp mesenchymal stem cell (DPSC) membranes contained CXCR2 carried DOX to form MOF-DOX@DPSCM	Killing activity and induced apoptotic effect of MOFDOX@DPSCM and specific targeting effect of DPSC membranes	MOF@DPSCM was specific to OSCC and could induce CAL-27 cell death in vitro and block CAL-27 tumor growth in vivo.	[73]
Zinc-based MOFs (Zn_4_O(C_8_H_5_NO_4_)_3_, IRMOF-3)	DOX and celecoxib (Cel)	MOFs were integrated with thermosensitive hydrogels to devise an injectable implant, and DOX Cel was coloaded into the system (DOX/Cel/ MOFs@Gel)	Toxic effects against OC cells of DOX and Cel	Exhibited a high capacity for drug loading, stable and pH-responsive release of dual drugs, and enhanced toxic effects on KB and SCC-9 cells in vitro. Displayed outstanding tumor inhibition efficacy in vivo, inducing tumor apoptosis and regulating tumor angiogenesis. Had relatively low systemic toxicity and no apparent damage to other organs.	[175]
Metal-organic framework material PCN-224	CQ (chemotherapy drug) and metal-organic framework material PCN-224 (PDT PS)	Autophagy-inhibiting photodynamic nano platform PCN-CQ@CCM coated with an OC cell membrane	PDT-generated ROS triggered the apoptosis pathway, as assessed by mitochondrial damage, and the released CQ further aggravated the ROS lethal pathway by effectively inhibiting the protective autophagic flux.	PCN-CQ@CCM could escape macrophage phagocytosis and adhere to tumor cells homologically, enhancing the retention and uptake of nanomaterials in the TME. After being activated with a 660 nm laser, the generated ROS triggered the apoptosis pathway through mitochondrial damage, and the released CQ further aggravated the ROS lethal pathway by effectively inhibiting the protective autophagic flux.	[190]
MSN	5-FU	MSNs loaded with 5-FU (MSN-5-FU) were prepared first. Subsequently, the outer membrane vesicles (OMVs) of *Escherichia coli* were collected to wrap MSN-5-FU and prepare OMVs-MSN-5-FU.	The antitumor effect of 5-FU	The OMVs-MSN-5-FU DDS could slow the drug release rate, significantly inhibit OSCC cell proliferation, and regulate cancer cell metastasis to cervical lymph nodes.	[176]
	MTH1 inhibitor (TH287) and multi-drug resistance protein 1 (MDR1) siRNA	TH287 and siRNA were loaded in a hyaluronic acid (HA)-based MSN	TH287 selectively inhibited the MTH1 protein in cells and MDR1 siRNA, inhibited or suppressed the gene expression of MDR1 in the cancer cells	Effectively controlled drug release and internalization in CAL-27 cells. The combination of TH287+MDR1 siRNA induced anticancer effects of tumor cells more effectively than TH287 alone. SiTMSN and HA-siTMSN significantly reduced tumor burden compared to untreated controls and free TH287.	[177]
	MDR1-siRNA and DOX	MSNs were modified by cationic polymer polyethylenimine (PEI) to obtain positive charges on the surface, which could enable the MSNP to carry MDR1-siRNA and DOX	MDR1-siRNA blocked MDR1 expression and DOX-induced apoptosis of cancer cells	Effectively reduced the expression of the MDR1 gene, induced apoptosis of KBV cells in vitro, and significantly reduced tumor size and slowed tumor growth in vivo compared to the control group.	[178]
Light-responsive MSNs	DOX (chemotherapy drug) and IR820, a new green cyanine dye (PTT agent)	Incorporate light-responsive MSNs as DOX carriers into the IR820/methylcellulose hydrogel networks	IR820-mediated photothermal effects and MSNs achieved self-degradation-controlled DOX release via the cleavage of diselenide bonds induced by ROS	Through the combination of chemotherapy and PTT, long-lasting synergistic antitumor effects could be obtained both in vivo and in vitro with less toxicity.	[191]
Multifunctional hyaluronic acid (HA) modified gold nanorods/mesoporous silica-based NPs	DOX hydrochloride (chemotherapy drug) and gold NPs (PTT agent)	Multifunctional HA-modified gold nanorods/mesoporous silica-based NPs loaded with DOX hydrochloride (DOX-AuNRs@mSiO_2_-HA)	Chemo-antitumor effect of DOX and PDT effect mediated by DOX-AuNRs@mSiO_2_-HA	Had excellent photothermal conversion efficiency in PTT. Combined chemotherapy-PTT therapy’s therapeutic effect was better than chemotherapy or PTT alone. NPs injected into CAL-27 tumor-bearing mice combined with NIR laser irradiation could accumulate rapidly in tumor sites and achieve complete tumor ablation without significant side effects on normal tissues.	[192]
Hollow mesoporous MnO_2_ nano-shells	DTX and CDDP	Hollow mesoporous MnO_2_ (H-MnO_2_) nano-shells were loaded with DTX and CDDP to form H-MnO_2_-PEG/TP nano-shells	H-MnO_2_-PEG/TP nano-shells decomposed in the acidic TME, releasing the loaded drugs and simultaneously attenuated tumor hypoxia and hypoxia-inducible factor-1α (HIF-1α) expression by inducing endogenous tumor hydrogen peroxide (H_2_O_2_) decomposition.	The proliferation, colony formation, and migration of CAL-27 and SCC-7 cells in the H-MnO_2_-PEG/TP group were significantly decreased, apoptosis was enhanced, and HIF-1α expression was down-regulated compared with the control group. The ratio of tumor uptake to normal organs in the H-MnO_2_-PEG/TP group was significantly higher than in the group without the NP, and tumor growth was partially delayed.	[180]
Fe_3_O_4_ Magnetic NPs	Therapeutic siRNAs targeting B-cell lymphoma-2 (BCL2) and Baculoviral IAP repeat-containing 5 (BIRC5)	Polyethyleneimine (PEI)-modified magnetic Fe_3_O_4_ NPs loading BCL2 and BIRC5	RNA interference triggered by siRNA	The NPs blocked siRNA in a concentration-dependent manner, and the NP-delivered siRNAs targeting BCL2 and BIRC5 significantly inhibited the viability and migration of Ca9-22 cells.	[179]
TiO_2_ NPs	A siRNA targeting HIF-1α (gene therapy drug) and ruthenium-based photosensitizer (Ru) (PDT PS)	TiO_2_@Ru@siRNA constructed from a ruthenium-based photosensitizer (Ru) modified-TiO_2_ NPs (NPs) loaded with siRNA of HIF-1α	TiO_2_@Ru@siRNA elicited photodynamic effects, which caused lysosomal damage, HIF-1α gene silencing, and OSCC cell elimination. TiO_2_@Ru@siRNA reshaped the immune microenvironment by downregulating key immunosuppressive factors, upregulating immune cytokines, and activating CD4^+^ and CD8^+^ T lymphocytes.	Inducing a photodynamic effect under visible light irradiation, effectively causing lysosome damage, HIF-1α gene silencing, and OSCC cell elimination. TiO_2_@Ru@siRNA-mediated PDT could significantly inhibit tumor growth and progression and enhance cancer immunity.	[193]
Fe_3_SO_4_ Magnetic NPs	CDDP	FA-mediated CDDP Fe_3_SO_4_ magnetic NPs (FA-CDDP-MNPs)	Cytotoxic effect of CDDP	A more significant inhibitory effect on OSCC than CDDP alone. Due to the introduction of FA, the targeting of FA-CDDPMNPs was improved, and the cytotoxicity was reduced.	[61]
CaCO_3_ NPs (CCNPs)	CDDP and Chrysin	Layer-by-layer of poly [di(sodium carboxyphenoxy)phosphazene] (PDCPP) and poly (diallyldimethyl ammonium chloride) (PDADMAC) deposited on the CaCO_3_ NPs (CCNPs) surface to form multi-layer NPs (MLNPs). CDDP and Chrysin were incorporated in the porous mineralized CaCO_3_ NP core and a polymeric shell	Cytotoxic effect of CDDP and chrysin-activated ROS production	Improved cytotoxic potential of MLNPs. Chrysin activated ROS production and eventually led to mitochondrial dysfunction. Buccal pouch carcinoma in the hamster model was significantly reduced. Dual-drug loaded MLNPs achieved 92% regressions of tumor volume compared to MLNPs loaded with CDDP alone.	[181]
Nanoscale graphene oxide (NGO)	DOX	PEG functionalized NGO carrying DOX, modified by tumor-specific peptide (HN-1) (DOX@NGO-PEG-HN-1)	Cytotoxic effect of DOX and targeting effect of HN-1 on OSCC	Significantly higher cellular uptakes and cytotoxicity in CAL-27 and SCC-25 cells when compared to free DOX. HN-1 showed considerable tumor-targeting and competition inhibition phenomena.	[65]
GQDs	Pt	A DDS based on Pt-loaded, polyethylene glycol-modified graphene quantum dots (GPt) via chemical oxidation and covalent reaction	GPt enhanced Pt accumulation in cells, which led to a notable increase of S-phase cell cycle arrest and apoptosis of OSCC cells	OSCC cells were sensitized to GPt under both normoxia and hypoxia conditions. GPt enhanced the accumulation of Pt in OSCC cells, resulting in a significant increase in S-phase cell cycle arrest and apoptosis of OSCC cells under normoxic and hypoxic conditions. GPt had a more substantial inhibitory effect on tumor growth and less systemic drug toxicity than free CDDP.	[182]
Highly-dispersive calcined HA NPs (nano-SHAP)	Zoledronic acid (ZA), CDDP, and carboplatin	Nano-SHAP was dissolved in distilled water, after which each drug was added and suspended	Antitumor activity of ZA, CDDP, and carboplatin	Nano-SHAP alone did not affect the proliferation of any cell line until a concentration of 1 μg/mL was reached. ZA-bearing nano-SHAP inhibited cell proliferation better than ZA alone. CDDP and carboplatin-bearing nano-SHAP had the same effect as these drugs alone	[183]

### 3.4. Other Types of Nano-DDSs

Other NPs, such as nucleic acid nanostructures and niosome nanocapsules, have been applied to form nano-DDSs for OC therapy [57,194]. Table 5 summarizes recent studies of other NPs used in nano-DDS formation for OC therapy.

**Figure 7:**
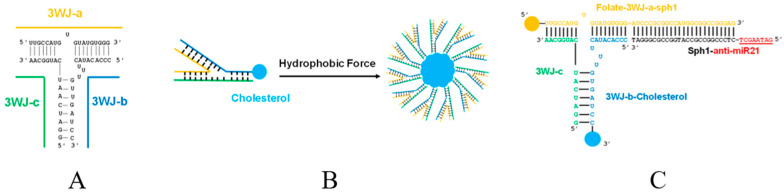
Design, construction, and characterization of RNA micelles. (**A**) 3WJ motif of pRNA from bacteriophage phi29 DNA packaging motor. (**B**) pRNA-3WJ micelle formation by hydrophobic force. (**C**) 2D structure of 3WJ/FA/anti-miR21 micelles. Reproduced with permission from ref. [57]. (Copyright 2019 ACS Nano).

Micelles formed by the assembly of amphiphilic peptides and polymerized nucleic acids can be used for drug delivery. These micelles have a core-shell structure comprising hydrophilic and lipophilic modules, which can encapsulate hydrophobic drugs inside and incorporate small interfering RNA as branches outside. Nucleic acid materials, including RNA, are also being explored as safe biomaterials for micelle formation due to their biocompatibility, structural programmability, and ease of alterations. Unlike polymeric NPs and LBNPs, nucleic acid-based NPs can be degraded by nucleases inherent in themselves, preventing toxicity associated with the delivery vehicles [195]. In a study by Yin et al. [57], RNA micelles were used to deliver anti-miRNA to human KB cancer cells. The 3WJ (motif derived from the pRNA of the bacteriophage phi29 DNA packaging motor)/FA/anti-miR21 micelles were composed of four strands: folate-3WJ-a-sph1, Sph1-anti-miR21, 3WJ-b-cholesterol, and 3WJ-c. They were synthesized using solid-phase synthesis or followed by a chemical reaction (Figure 7). These RNA micelles carrying anti-miR21 demonstrated strong binding and internalization to human KB cancer cells, leading to inhibition of oncogenic miR21 function, enhanced expression of pro-apoptotic factors, and induction of cell apoptosis. Animal experiments also showed effective tumor targeting and inhibition by these RNA micelles in xenograft models.

Different from the phospholipid components of liposomes, niosome nanocapsules are carriers formed by the accumulation of non-ionic surfactants in the aqueous medium, with a vesicle-like bilayer construction consisting of hydrophilic and hydrophobic segments [196]. Drug-loaded niosomes can enhance the pharmacokinetics of the encapsulated drug, leading to improved therapeutic effects and reduced side effects. A study by Fazli et al. [194] investigated the prophylactic effect of Cur niosomes, prepared using the thin-film hydration method, in both topical (mouthwash) and systemic (injectable) forms on induced OC in rats. The results revealed that injectable Cur niosomes effectively prevented the development of severe forms of dysplasia. Furthermore, the culture medium of KB OC cells showed that Cur-loaded niosomes exhibited greater efficacy in inhibiting cancer cell growth and necrosis compared to free Cur.

### 3.5. Clinical Applications of Nano-DDSs in OC Therapy

Recent clinical trials on nano-DDSs in OC therapy [197] are demonstrated in Table 6. It is clear that the nano-DDSs applied in OC therapy are still in the research and development stage, and to date, no nano-DDSs have been approved for clinical treatment of OC. Despite some advances in nano-DDSs in OC therapy, most OC therapies still employ traditional treatment methods such as surgery, radiation, and chemotherapy. Researchers and pharmaceutical companies are trying to develop and evaluate nano-DDSs for various OC therapies to improve drug targeting, bioavailability, and therapeutic effectiveness while reducing drug toxicity.

## 4. Conclusions

NPs have been extensively studied for their potential use in the nano-DDS formation to treat OC. These nano-DDSs can act as carriers for chemotherapy drugs and therapeutic agents of light stimulus-responsive therapies, leading to improved clinical outcomes and quality of life for OC patients. This article provides an overview of recent advances in the use of nano-DDSs for treating OC, highlighting the challenges and progress achieved using various nano-DDSs.

Nano-DDSs can be formed by loading NPs with antitumor drugs. By modifying these NPs with targeting agents, OC cells can be effectively targeted with the loaded drugs, minimizing damage to healthy cells as well as exhibiting site-specific delivery behavior. The primary types of NPs studied for treating OC include polymeric NPs, lipid-based NPs, and inorganic NPs. These nano-DDSs improve the bioavailability of anti-OC drugs through various mechanisms. They can enhance the stability of the drugs, facilitate the crossing of biological barriers, increase drug solubility, and enable targeted delivery and controlled release. These mechanisms ultimately improve the therapeutic efficiency of treating OC by reducing the drug dose, administration frequency, and drug resistance.

It is worth noting that although most nano-DDSs mentioned in this review showed positive results in treating OC, their clinical use for treating OC is currently limited. To enhance the translation of these technologies into clinical practice, it is essential to clarify the uptake and retention mechanisms of NPs in vivo and address the fundamental physiological differences between humans and experimental animals. Additionally, some nano-DDSs may be toxic and pose potential risks to oral tissue and overall health, which restricts their clinical application. Therefore, it is necessary to ensure that nano-DDSs are made from biocompatible and non-toxic materials, undergo comprehensive toxicity assessments, and modify their surface properties to minimize toxicity. Nano-DDSs face challenges in effectively targeting OC cells, resulting in suboptimal drug delivery to the targeted site. To improve targeting efficiency, specific ligands or antibodies can be used to identify OC cells and enhance the binding affinity and specific interaction between nano-DDSs and cancer cells. Some nano-DDSs may exhibit slow or inefficient biodegradation and clearance from the body, leading to potential accumulation and long-term effects. To solve this question, it is recommended to introduce biodegradable materials or modify nano-DDSs to promote their degradation and efficient clearance from the body. Moreover, Nano-DDSs may have limitations in drug loading capacity and controlled release rate. To overcome these challenges, it is suggested to optimize the nano-DDSs by means of adopting porous structures or encapsulation techniques to increase drug loading and fine-tuning their release dynamics by adjusting the size, shape, or surface properties of the nanoparticles. By addressing these limitations and ensured safety, improved targeting efficiency, enhanced biodegradability and clearance, and optimized loading and release kinetics of the drugs, the clinical application of nano-DDSs in OC therapy can be promoted. Therefore, significant efforts are required to develop more effective nanomedicines for the treatment of OC. It is crucial to conduct precise clinical trials to validate the availability of nanotechnology based on promising in vitro and in vivo studies, thereby accelerating their clinical applications.

## Figures and Tables

**Table 1:** Summary of ligands and targeted receptors overexpressed in OC cells.

Ligand	Target Receptor	Ref.
Folic acid (FA)	FA receptor	[56,57,58,59,60,61,62]
Protein corona-modulating Tf2 peptide	Transferrin receptor (TfR)	[63]
HN-1, a 12-amino acid peptide	HN-1 receptor	[64,65]
Anti Programmed death-ligand 1 (PD-L1) antibody	PD-L1	[66,67]
α-tocopherol	α-tocopherol receptor	[68]
Fucoidan	Scavenger-A receptors (SR-A), L-selectin, Toll-like receptors, and PD-L1	[69]
Antibodies specific for matrix metalloproteinase-1 (MMP-1)	MMP-1	[70]
pH-sensitive H-peptide	Epidermal growth factor receptor (EGFR)	[71]
Anti-Her-2 (human epidermal growth factor receptor 2) nanobody	Her-2	[72]
Dental pulp mesenchymal stem cell (DPSC), which expresses the CXCL8 binding receptor, CXCR2	Chemokine CXCL8	[73]
Chemokine SDF-1	CXC chemokine receptor 4 (CXCR4)	[74]
Shiga Toxin-B	Globotriaosylceramide receptor (GB3)	[75]
Fibroblast activation protein (FAP)-targeted peptide chains	FAP	[76]
AE105 (H-Asp-Cha-Phe-(d)Ser-(d)Arg-Tyr-Leu-Trp-SerOH) peptide	Urokinase plasminogen activator receptor (uPAR)	[77]

**Table 5:** Studies on other NPs applied for nano-DDS formation in OC therapy.

Nanocarrier	Active Agent	Assembly Method	Mechanism	Advantages	Ref.
RNA Micelles	Anti-miRNA	The phi29 packaging RNA three-way junction(pRNA-3WJ) was used as a scaffold to construct micelles. An oligo with 8 nt locked nucleic acid (LNA) complementary to the seed region of microRNA21(miR21) was included in the micelles as an interference molecule for cancer inhibition	Internalization of anti-miRNA inhibited the function of oncogenic miR21, enhanced the expression of the pro-apoptotic factor, and induced cell apoptosis	The RNA micelles carrying anti-miR21 showed strong binding and internalization to cancer cells, inhibited the function of oncogenic miR21, enhanced the expression of the pro-apoptotic factors, and induced cell apoptosis. Animal experiments revealed effective tumor targeting and inhibition of RNA micelles in xenograft models.	[57]
Nano-niosomes	Cur	Niosomes containing Cur were prepared by the thin-film hydration method	Cur counteracted the effects of 4-NQO to cause cancer in the oral tissue through its antioxidant properties	The use of injectable Cur niosomes significantly prevented the development of severe forms of dysplasia. According to the results obtained from the culture medium of KB OC cells, Cur-loaded niosomes were found to be significantly effective in inhibiting the growth and necrosis of cancer cells compared with free Cur.	[194]

**Table 6:** Recent clinical trials on nano-DDSs in OC therapy [197].

ClinicalTrials.Gov Identifier	Type of Cancer	Nanocarrier	Active Ingredients	Phase	Recruitment Status
NCT05456022	Tongue Squamous Cell Carcinoma Cell Line	PLGA-PEG NPs	QRC	Phase Ⅱ	Not yet recruiting
NCT01847326	Squamous Cell Carcinoma of the Hypopharynx; Squamous Cell Carcinoma of the Lip and Oral Cavity	HSA NPs	PTX	Phase I	Active, not recruiting

## Data Availability

Not applicable.
